# Thermoelectric Materials and Devices

**DOI:** 10.3390/mi17040437

**Published:** 2026-03-31

**Authors:** Ruomeng Huang

**Affiliations:** School of Electronics and Computer Science, University of Southampton, Southampton SO17 1BJ, UK; r.huang@soton.ac.uk

Thermoelectric (TE) materials and devices enable the direct conversion between thermal and electrical energy, offering solid-state solutions for power generation and cooling without moving parts or working fluids [1]. This unique capability has positioned thermoelectric technologies as promising candidates for a wide range of applications, including waste heat recovery, energy harvesting in low-power electronic systems, and localised thermal management [2]. With increasing global emphasis on energy efficiency and sustainability, thermoelectrics continue to attract significant research interest across materials science, physics, and engineering.

Early developments in thermoelectric research primarily focused on bulk materials, with performance improvements pursued through optimisation of the dimensionless figure of merit, *ZT*. Strategies such as alloying, doping, and nanostructuring have led to notable enhancements in thermoelectric properties [3,4]. However, the translation of high material performance into practical devices remains nontrivial, as device efficiency and reliability are strongly influenced by factors beyond intrinsic material properties, including thermal and electrical interfaces, mechanical stability, and fabrication constraints [5]. In recent years, thermoelectric research has expanded beyond material-centric studies toward a more holistic consideration of devices and systems. This shift has been driven by increasing demand for thermoelectric solutions across emerging application areas, including distributed sensing, wearable electronics, and localised cooling of electronic components [6]. In these contexts, device architecture, integration strategies, and operating environment often play a decisive role in determining overall performance, sometimes outweighing gains achieved at the material level alone. Concurrently, advances in fabrication technologies and modelling approaches have enabled increasingly sophisticated thermoelectric device designs, facilitating the exploration of new form factors, including thin-film, flexible, and hybrid systems [7]. Collectively, these developments highlight the importance of co-optimising materials, device structures, and thermal management strategies to meet the requirements of real-world applications.

Thermoelectric technologies are currently experiencing renewed momentum, driven not only by advances in materials science but also by increasing demand for energy-efficient thermal management and distributed energy harvesting in emerging electronic systems. In this context, there is a need to examine thermoelectric research from a broader perspective that connects materials innovation with device integration and system-level optimisation. Motivated by these developments, this editorial highlights advances across these interconnected areas and outlines emerging research directions that may shape the next stage of thermoelectric device development. By integrating insights from materials development, device engineering, and application-driven system design, this article aims to offer a concise perspective on the evolving thermoelectric research landscape.

Recent progress in thermoelectric (TE) materials has been driven by the need to balance three coupled targets: increasing the power factor, suppressing lattice thermal conductivity, and maintaining stability and manufacturability in device-relevant forms. Across material families, a common theme is the use of band and defect engineering (e.g., carrier concentration control, band convergence, resonant levels), alongside phonon scattering strategies (e.g., point defects, nanoprecipitates, grain boundary engineering) to decouple electrical and thermal transport as far as possible [8,9]. Chalcogenide thermoelectrics remain a central materials class due to their inherently low lattice thermal conductivity and strong tunability through alloying and defect control. Recent high-impact studies on SnSe illustrate how structural and electronic optimisation can simultaneously enhance carrier transport and maintain low thermal conductivity, underscoring the continuing relevance of chalcogenide design principles [10,11].

A particularly important direction over the last five years has been the rapid maturation of tellurium-free, magnesium-based thermoelectrics, motivated by elemental abundance, cost, and sustainability considerations. Among these, Mg_3_(Sb,Bi)_2_ alloys have emerged as leading mid-temperature candidates, supported by a growing body of work spanning materials optimisation and device parameters [12,13]. Detailed composition-adjustment studies demonstrate that varying the Sb/Bi ratio significantly modifies band dispersion and carrier effective mass, enabling optimisation of the Seebeck coefficient and electrical conductivity through controlled carrier concentration tuning [13]. Aliovalent doping further optimises carrier density and mobility, mitigating ionised impurity scattering while preserving high power factors. From a thermal transport perspective, Mg_3_(Sb,Bi)_2_ benefits from intrinsically low lattice thermal conductivity arising from anharmonic bonding and complex crystal dynamics. This behaviour can be further enhanced via nanotechnological approaches, including grain refinement, defect clustering, and the introduction of nanoscale secondary phases that promote hierarchical phonon scattering across multiple length scales. Complementing the Mg_3_(Sb,Bi)_2_ family, MgAgSb continues to be actively developed for near-room-temperature operation. Recent reviews highlight that phase-boundary control, microstructural engineering, and controlled sintering processes are critical for stabilising the desired α-phase while minimising grain-boundary resistance [14]. Nanostructural control—including refined grain size, engineered interfaces, and second-phase inclusions—not only enhances phonon scattering but also improves mechanical robustness and chemical stability, thereby addressing practical deployment challenges such as oxidation sensitivity and interfacial degradation. Collectively, these strategies illustrate how advanced materials design, informed by both electronic band engineering and nanoscale structural control, enables systematic enhancement of thermoelectric performance in Mg-based systems.

While materials development remains essential, the translation of thermoelectric performance into practical impact is increasingly determined at the device and system levels. Application requirements vary widely: wearable and IoT harvesters must operate under small temperature gradients, conform to soft form factors, and maintain stable output under motion and changing ambient conditions [15,16]; microelectronic cooling targets rapid thermal response and hotspot-level control with tight constraints on footprint, thermal interfaces, and power density [17]; and industrial waste-heat recovery prioritises long-term stability, scalable module assembly, and cost-effective heat-exchanger integration [18]. These diverse use cases have driven a shift from “materials-first” benchmarking toward integration-aware metrics, including power density, load matching, interface thermal resistance, mechanical reliability, and power management compatibility. Most thermoelectric systems still employ the familiar p–n leg module architecture, but modern implementations increasingly diversify along two directions. First, micro- and thin-film devices seek compatibility with manufacturing workflows and thermal management stacks, often requiring careful co-design of leg geometry, metallization, and interconnect routing to minimise parasitics and accommodate packaging constraints [19]. Second, flexible and hybrid architectures (printed, textile-integrated, or nanocarbon-based) aim to maintain electrical continuity while enhancing heat transfer and mechanical durability in soft systems [20,21]. A recurring integration bottleneck across all architectures is that the effective device temperature gradient is often far smaller than the available environmental gradient, due to thermal contact resistances and imperfect heat spreading—making thermal interface engineering as important as the thermoelectric material itself [22].

As thermoelectric technologies move closer to practical deployment, device-level design and optimisation have become as critical as materials selection in determining overall performance. Even for a given thermoelectric material system, achievable efficiency and power output can vary substantially depending on device geometry, operating conditions, and system integration. Consequently, recent research has increasingly emphasised optimisation frameworks that explicitly consider thermal, electrical, and mechanical constraints at the device and module levels. The performance of a thermoelectric device is governed by a set of tightly coupled parameters, including leg geometry (length, cross-sectional area, and aspect ratio), electrical and thermal contact resistances, packing density, and heat-spreader configuration. These parameters directly influence internal electrical resistance, heat leakage, and the effective temperature gradient across the active material. Therefore, optimisation involves balancing competing objectives, such as maximising power density while limiting parasitic losses or enhancing efficiency without compromising mechanical robustness and manufacturability. In practice, the “optimal” design is often application-specific, reflecting the distinct boundary conditions and constraints associated with wearable energy harvesting, microelectronic cooling, or large-scale waste-heat recovery.

Early device optimisation efforts relied primarily on analytical models, which provide valuable physical insight and scaling relationships between material properties, geometry, and performance metrics [23]. Such models remain useful for identifying dominant loss mechanisms and for guiding initial design choices. However, as device architectures and boundary conditions have become more complex, numerical methods, particularly finite-element modelling (FEM), have become the dominant tools for thermoelectric device design. FEM-based approaches enable coupled electrothermal simulations that capture non-uniform temperature fields, contact resistances, and realistic heat-transfer conditions, making them well suited for evaluating microstructured, thin-film, and hybrid thermoelectric devices [24,25]. While numerical simulation has significantly expanded thermoelectric design capability, exhaustive parameter sweeps quickly become computationally prohibitive as the dimensionality of the design space increases. This limitation has motivated growing interest in data-driven and machine-learning-enabled optimisation strategies, which aim to accelerate exploration of complex design spaces while preserving physical relevance [26]. Machine learning (ML) approaches are increasingly used not only to speed up optimisation, but also to reveal non-intuitive design trends and trade-offs that may be difficult to identify through conventional analysis. In high-dimensional optimisation problems—where geometric parameters, contact resistances, material properties, and operating conditions interact nonlinearly—the computational cost of exhaustive parametric sweeps grows exponentially. Surrogate models trained on physics-based simulation data can approximate performance landscapes with significantly reduced evaluation time, enabling rapid identification of near-optimal configurations. When integrated with evolutionary algorithms or Bayesian optimisation frameworks, ML-driven approaches can efficiently navigate complex design spaces while maintaining consistency with thermodynamic constraints. Representative examples include the use of regression models, neural networks, and evolutionary algorithms to optimise leg geometry, contact configurations, and operating conditions under realistic boundary constraints [27,28]. As thermoelectric devices continue to diversify in form factor and application, such hybrid physics–data approaches are expected to play an increasingly important role in device- and system-level optimisation.

Despite significant advances in thermoelectric materials, device integration, and optimisation strategies, several challenges continue to limit the widespread deployment of thermoelectric technologies. A persistent issue is the gap between laboratory-scale performance and system-level efficiency under realistic operating conditions, where thermal interfaces, parasitic losses, and environmental variability often dominate device behaviour. In addition, achieving scalable, reliable, and cost-effective manufacturing remains a key hurdle, particularly for devices that combine high performance with complex architectures or flexible form factors. Looking ahead, further progress is likely to depend on co-design approaches that integrate materials development, device architecture, and system-level considerations from the outset. Advances in modelling and data-driven optimisation are expected to play an increasingly important role in navigating complex design spaces and accelerating translation from concept to application. As thermoelectric devices continue to diversify across applications, research that bridges fundamental understanding and practical integration will be essential for realising their full technological potential.
